# Response to Perinatal Psychosis in West Essex During COVID-19 Pandemic

**DOI:** 10.1192/bjo.2022.375

**Published:** 2022-06-20

**Authors:** Hesham Abdelkhalek, Thando Sibindi, Vivienne Harris, Manal El-Maraghy

**Affiliations:** Essex Partnership University NHS Foundation Trust, Essex, United Kingdom

## Abstract

**Aims:**

The aim of this audit is to look at the presentation of women who were pregnant or less than one-year post-partum presenting with psychotic symptoms in the A&E Department, general hospital and calls to crisis line, particularly with the fact that the pandemic impact remains on the nation. Our aim was to ensure that all referred patients were assessed within the first 24 hours, all the assessments were completed face-to-face, a biopsychosocial assessment was completed for each patient and an outcome was agreed on and clearly documented in the notes.

**Methods:**

All referrals to West Essex access and assessment team from the A&E department, the general hospital and calls logged to crisis line were included. Data were collected prospectively over a six-month period from mid-November 2020 to mid-April 2021. For the purpose of this audit, an identification form was designed and disseminated to access and assessment and crisis teams to identify illegible patients. Our data collectors then used the main audit tool to gather the data.

**Results:**

In total, our sample included sixteen patients who met our criteria over the six months period. There was only one patient who was out of area. Most of the patients were of white British ethnicity (ten out of sixteen) and other six patients were five white other and one of Asian origin. The mean maternal age in our sample was 27.3 years old and the majority of the referrals came from the labour ward in Princess Alexandra hospital (57%). The two main outcomes of our audit were to check the response time and the way the initial assessment was carried over. Our results show that the team responded to all referrals on the same day with no delays. All the assessments were carried out in a face-to-face fashion in the general hospital apart from one assessment that came through the crisis line and this was carried out in the patient's home.

**Conclusion:**

From our data we can identify that the access and assessment team met the standards we set for this audit. This fulfills the recommendations of MBRRACE-report and the RCPsych. One of our recommendations was to provide educational sessions to the emergency department in the general hospital to raise awareness on psychotic presentation during perinatal period.

